# Deciphering the heterogeneous glucosinolates composition in leaves and seeds: strategies for developing *Brassica napus* genotypes with low seed glucosinolates content but high leaf glucosinolates content

**DOI:** 10.1186/s43897-025-00147-1

**Published:** 2025-05-01

**Authors:** Mengxin Tu, Wenxuan Guan, Antony Maodzeka, Hongyu Zhou, Zi Zhang, Tao Yan, Shuijin Hua, Lixi Jiang

**Affiliations:** 1 Institute of Crop Science, Zhejiang University, 866 Yu-Hang-Tang Road, Hangzhou, 310058 China; 2 Zhejiang Academy of Agricultural Sciences, Desheng Zhong Road 298, Hangzhou, 310022 China

**Keywords:** *Brassica napus*, Glucosinolates, Genome-wide association study, Transcriptome, Differentially expressed genes, Selective-sweep analysis

## Abstract

Rapeseed cakes with low glucosinolates content (GC) possess high feeding value. However, the pursuit of low-GC seeds has inadvertently resulted in a reduction of GC in leaves, making plants more susceptible to stress and lowering their nutritional quality. Therefore, it is imperative to disrupt the tight association between GC in these two tissues and ultimately develop genotypes with low-GC seeds but high-GC leaves. The distinct mechanisms underlying glucosinolate (GSL) synthesis in these two tissues remain unclear. Here, we discovered that aliphatic and aromatic GSLs, rather than indole GSLs, contribute to the positive correlation between GC in seeds and leaves. We performed selective-sweep analyses and identified the genomic footprints left after decades of intense selection for low-GC seeds. By conducting genome-wide association studies and analyzing differentially expressed genes in high- and low-GC seeds and leaves, we compiled lists of distinct genes involved in GSL synthesis in leaves and seeds separately. In particular, *BnMYB28* plays a key role in regulating GC in both seeds and leaves. Selection and manipulation of *BnaC09.MYB28* would affect GC in both tissues. However, downregulation of *BnaA02.MYB28* and/or *BnaC02.MYB28* would likely reduce GC in seeds without causing a concurrent reduction in GC in leaves.

**Supplementary Information:**

The online version contains supplementary material available at 10.1186/s43897-025-00147-1.

## Core

This study identifies distinct gene lists involved in GSL synthesis in leaves and seeds separately and demonstrates that aliphatic and aromatic GSLs, rather than indole GSLs, drive the positive correlation between GC in both tissues. In particular, different homologs of *BnMYB28* regulate GC in seeds and leaves through distinct mechanisms.

## Gene & Accession Numbers

The genes *BnaC09.MYB28* (*BnaC09G0066500ZS*), *BnaA02.MYB28* (*BnaA02G0394700ZS*), and *BnaC02.MYB28* (*BnaC02G0527500ZS*) were analyzed in this study. The raw reads for the rapeseed accessions have been deposited in the National Center for Biotechnology Information (NCBI) public database under SRP155312 (https://www.ncbi.nlm.nih.gov/sra/SRP155312) and in the China National Center for Bioinformation (NGDC) under CRA001854 (https://ngdc.cncb.ac.cn/gsa/browse/CRA001854).

## Introduction

Rapeseed (*Brassica napus* L., 2n = 38, AACC) is of significant agricultural importance in many parts of the world as one of the foremost oilseed crops. Early, unimproved rapeseed varieties accumulated high levels of undesirable compounds, such as glucosinolates (GSLs) in seeds, significantly affecting the use of the resulting cake as animal feed and limiting the economic value of rapeseed products. This breeding breakthrough enabled the production of high-quality rapeseed meal with reduced GSL content (GC), positioning it as the second most widely traded protein ingredient after soybean meal (Wanasundara et al. 2016).

GSLs are a class of specialized metabolites found in plants belonging to the order Brassicales (Sugiyama et al. 2021). Approximately 150 categories of GSLs have been identified, all sharing a common chemical structure comprising a β-D-thioglucose group, a sulfonated oxime group, and an amino acid-derived R group (Akram et al., 2021). GSLs can be classified into three main types: aliphatic, aromatic, and indole GSLs (Wittstock and Halkier 2002). GSLs and their hydrolysis products exhibit diverse biological functions and significantly impact the quality of rapeseed cake. However, research has indicated that GSLs present in plant vegetative organs play a favorable role in enhancing plants' resilience against various forms of environmental stress and adversity (Liu et al. 2021; Qin et al. 2023). Moreover, some GSLs have been found to possess anticancer and immunosuppressive properties that are beneficial to human health (Zhou et al. 2016).

The biosynthesis of glucosinolates (GSLs) can be broadly divided into three stages: side chain elongation, core structure formation, and modification of the R side chain (Barco and Clay 2019). Various families of enzymes and transcription factors (TFs) are involved in these processes. Among the key genes regulating GSL synthesis in *Brassicaceae* plants, *MYB28* stands out as a notable regulator. In rapeseed, *MYB28* has multiple copies, some of which are strongly associated with glucosinolate content (GC) (Schilbert et al. 2022). Overexpression of *BnaA09.MYB28* in transgenic *Arabidopsis thaliana* significantly increased the abundance of leaf aliphatic GSLs, including the predominant 4-methylsulphinylbutyl-glucosinolate (4MSOB) (Long et al. 2016). *BnaC02.MYB28* was identified through a double haploid population derived from a cross between two rapeseed accessions with varying seed GC (Liu et al., 2020a). This gene is posited as the likely candidate underlying the major quantitative trait locus (QTL), *qGSL-C2*, and has been confirmed as a positive regulator of seed GC by forming homodimers, interacting with *BnaMYC3*, and directly activating the expression of GSL biosynthesis genes (Zhou et al., 2023). Additionally, *BnaC09.MYB28* has been identified as the candidate gene for the significant QTL *qGSL.C09.1* through a genome-wide association study (GWAS) on seed GC (Tan et al. 2022). Elevated transcript levels of *BnaC09.MYB28* correlate with enhanced GSL biosynthesis in seeds (Wei et al. 2019). More recently, Schilbert et al. (2022) reported a 4 bp insertion in *BnaC09.MYB28* that demonstrates a significant association with a reduction in GC in seeds. This was determined by investigating a segregating F2 population to identify genomic intervals and candidate genes associated with GC in *B. napus* seeds. The significance of *MYB28* in GSL biosynthesis extends beyond *Brassica napus* to other species within the genus, including *Brassica rapa*, *Brassica oleracea*, and *Brassica juncea* (Seo et al. 2016; Augustine et al. 2013; Neequaye et al. 2021).

The origin of low-GC rapeseed is commonly attributed to the introduction of a single variety known as the Polish ‘*Bronowski*’, which resulted in a narrowing of genetic diversity among low-GC genotypes (Finlayson et al., 1973). The process of targeted breeding selection leads to the fixation of beneficial alleles within a population, consequently reducing genetic variation among neighboring nucleotide sequences. The measurement of selection pressure can be assessed using *F*_*ST*_ (fixed coefficient of differentiation), which represents the genetic differentiation index and provides information on polymorphism data between and within different subpopulations (Stephan 2019). On the other hand, GWAS serve as powerful tools to identify genes associated with variations in specific traits within a genetic population. Unlike hypothesis-driven approaches, GWAS are unbiased and can reveal novel genetic associations even when the functions of the implicated genes are unknown, overcoming challenges posed by incomplete knowledge and unidentified factors (Kitsios and Zintzaras 2009).

Researchers have observed a concurrent decrease in GC not only in the seeds but also in the nutritional tissues of 'double low' rapeseed cultivars. This reduction in GC within vegetative tissues carries significant implications. It compromises disease and pest resistance, rendering the plants more vulnerable to stress during growth, and negatively impacts the nutritional value of the leaves. This is particularly concerning given the potential use of rapeseed plants as green fodder or as vegetables in human diets (Becker and Juvik 2016). Therefore, it is crucial to disrupt the strong association between GC in these two tissues in order to develop genotypes characterized by low-GC seeds and high-GC leaves. However, the specific mechanisms governing GSL synthesis in these two tissues remain unclear.

In this study, our objective was to investigate the genetic characteristics resulting from extensive selection for low-GC seeds over the past half-century. We accomplished this by analyzing overall GC as well as the levels of individual GSL species in both seeds and leaves of a genetic population consisting of 235 rapeseed accessions. To achieve our goal, we employed a combination of selective-sweep analysis (SSA), GWAS, and transcriptome analysis. Through these approaches, we unraveled the molecular mechanisms responsible for the synthesis of distinct GSLs present in leaves and seeds. Furthermore, we identified crucial genes that should be selectively targeted or avoided, with the intention of preserving low-GC seeds while simultaneously augmenting GC levels in vegetative tissues.

## Results

### The differential GSL spectra between leaves and seeds exhibit overlapping features

**Fig. 1:**
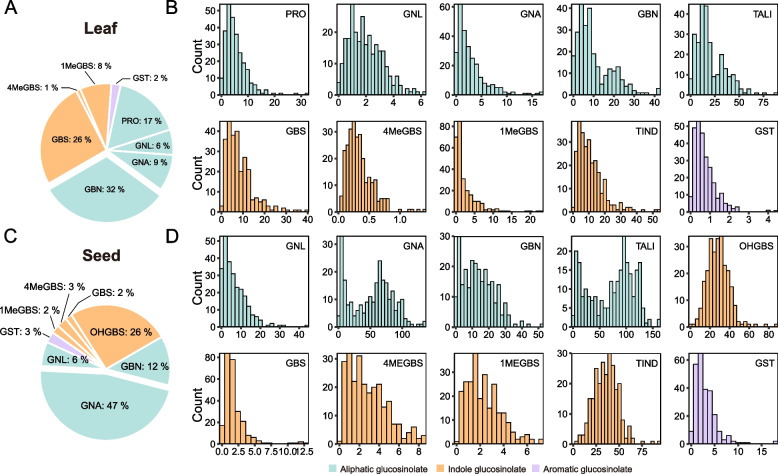
Comparison of total and individual GSL species between seeds and leaves in the population. **A** Pie chart illustrating the distribution of individual GSL species in leaves. **B** Count of accessions in different GC categories for various types of GSLs and total GC in leaves. **C** Pie chart demonstrating the distribution of individual GSL species in seeds. **D** Count of accessions in different GC categories for various types of GSLs and total GSLs in seeds. Aliphatic GSLs are represented by green, indole GSLs by orange, and aromatic GSLs by purple. Specific GSL abbreviations and their corresponding full names are as follows: PRO (Progoitrin), GNA (Gluconapin), GNL (Gluconapoleiferin), GBN (Glucobrassicanapin), GBS (Glucobrassicin), OHGBS (4-Hydroxyglucobrassicin), 4MeGBS (4-Methoxyglucobrassicin), 1MeGBS (1-Methoxyglucobrassicin), GST (Gluconasturtiin), TALI (Total aliphatic GSL), TIND (Total indole GSL)

The GC in the seeds and leaves of the accessions was quantified using High-Performance Liquid Chromatography with Diode Array and Ultraviolet detection (HPLC–DAD/UV) (Table S1). In our study, a total of nine GSL species were identified, including four aliphatic GSLs (progoitrin [PRO], gluconapin [GNA], gluconapoleiferin [GNL], and glucobrassicanapin [GBN]), four indole GSLs (glucobrassicin [GBS], 4-hydroxyglucobrassicin [OHGBS], 4-methoxyglucobrassicin [4MeGBS], and neoglucobrassicin [1MeGBS]), and one aromatic GSL (gluconasturtiin [GST]) (Table 1). The aliphatic GSL PRO was exclusively detected in leaves, while OHGBS was only found in seeds. However, the remaining seven GSL species were detectable in both leaf and seed tissues. Notably, the proportions of individual GSLs in the two categories, aliphatic and indole GSLs, exhibited significant variation between the two tissues (Fig. 1 A, C). In leaves, the most abundant GSLs were the aliphatic GBN and the indole GBS, while in seeds, the most abundant GSLs were the aliphatic GNA and the indole OHGBS. These distinct GSL composition profiles in leaves and seeds led to the development of a set of 20 parameters for phenotypic characterization. These parameters included eight GSL species (PRO, GNL, GNA, GBN, GBS, 4MeGBS, 1MeGBS, and GST) in leaves and eight GSL species (GNL, GNA, GBN, OHGBS, GBS, 2MeGBS, 1MeGBS, and GST) in seeds, as well as the total aliphatic GSLs (TALI) and total indole GSLs (TIND) in both tissues. The concentration distribution of these GSLs exhibited extensive and continuous variation among the individual accessions of the population (Fig. 1 B, D; Table S2).

**Table 1:** The basic information of glucosinolates identified in *Brassica napus*

Type	Name	Abbreviations	Systematic R side chain	Organ
Aliphatic C4	Progoitrin	PRO	(2R)−2-Hydroxy-3-butenyl	Leaf
	Gluconapin	GNA	3-Butenyl	Leaf, seed
Aliphatic C5	Gluconapoleiferin	GNL	2-Hydroxy-pent-4-enyl	Leaf, seed
	Glucobrassicanapin	GBN	Pent-4-enyl	Leaf, seed
Indole	Glucobrassicin	GBS	3-Indolylmethyl	Leaf, seed
	4-Hydroxyglucobrassicin	OHGBS	4-Hydroxy-3-indolylmethyl	Seed
	4-Methoxyglucobrassicin	4MeGBS	4-Hydroxy-3-indolylmethyl	Leaf, seed
	Neoglucobrassicin	1MeGBS	4-Hydroxy-3-indolylmethyl	Leaf, seed
Aromatic	Gluconasturtiin	GST	2-Phenethyl	Leaf, seed

### The aliphatic and aromatic GSLs, rather than indole GSLs, contribute to the strong correlation of GC between leaves and seeds

**Fig. 2:**
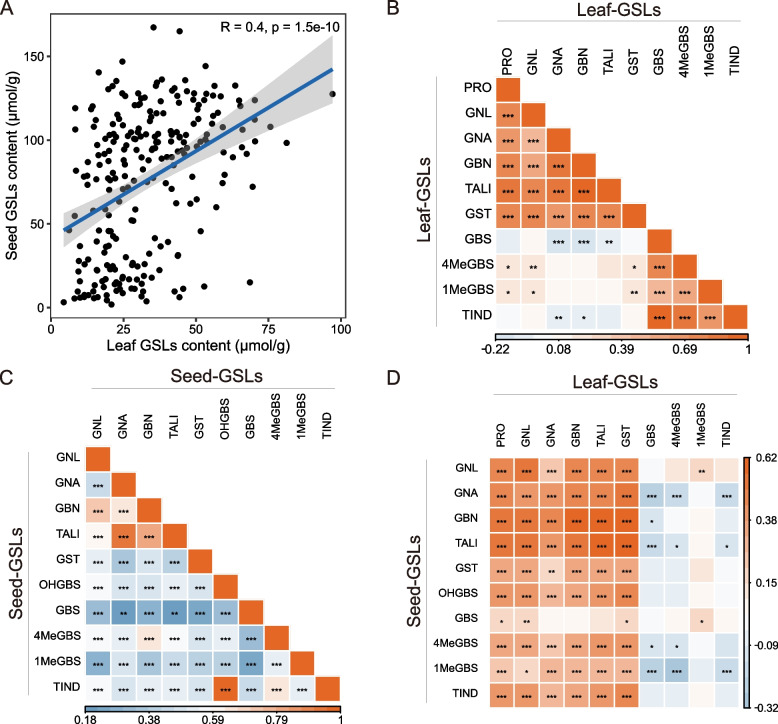
Correlation analysis of total and individual GSL species between leaves and seeds. **A** Correlation between total GC in leaves and seeds. **B**, **C** Correlation between individual GSL content in leaves (B) and seeds (C). **D** Correlation of individual GSL content between leaves and seeds. Abbreviations for specific GSL species and their corresponding full names are as follows: PRO (Progoitrin), GNA (Gluconapin), GNL (Gluconapoleiferin), GBN (Glucobrassicanapin), GBS (Glucobrassicin), OHGBS (4-Hydroxyglucobrassicin), 4MeGBS (4-Methoxyglucobrassicin), 1MeGBS (1-Methoxyglucobrassicin), GST (Gluconasturtiin), TALI (Total aliphatic GSL), TIND (Total indole GSL)

We conducted an analysis to examine the correlations among the parameters mentioned above in leaves and seeds. Overall, we observed a significant positive correlation in the total GC content between seeds and leaves (Fig. 2A). Within the leaves, there was a strong correlation among the concentrations of individual GSLs within the same GSL category. However, the correlation between different categories, namely aliphatic and indole GSLs, was non-significant (Fig. 2B). In contrast, the different categories of GSLs in seeds displayed generally weak but still significant positive correlations (Fig. 2C). The aliphatic GSLs in seeds, whether considered collectively (TALI) or individually, showed a significant positive correlation with the corresponding parameters in leaves (Fig. 2D). The correlation coefficient of TALI between seeds and leaves reached 0.56 (*p* < 0.001). On the other hand, the total indole GC (TIND) in seeds and leaves did not show a significant positive correlation (Fig. 2D); in particular, the concentrations of certain indole GSL components, such as 4MeGBS and 1MeGBS, exhibited negative correlations or no significant correlations between leaves and seeds (Fig. 2D). The content of the aromatic GSL, i.e., GST, in seeds and leaves was significantly correlated. Furthermore, the concentration of GST in seeds showed a positive correlation with the contents of aliphatic GSLs, such as PRO, GNL, GNA, and GBN (Fig. 2D). These results suggest that the positive correlation in the total GC between leaves and seeds is primarily driven by the aliphatic and aromatic GSLs, while indole GSLs may be synthesized differently in leaves and seeds.

Additionally, we conducted a comparative analysis of GC in leaves and seeds among 30 accessions with the highest seed GC and 30 accessions with the lowest seed GC (Fig. S1). Regarding the leaf GSL composition of these accessions, we observed relatively high concentrations of aliphatic GSLs (PRO, GNL, GNA, GBN) and the aromatic GST in the high-GC seed (H-HC-S) accessions compared to the low-GC seed (L-GC-S) accessions (Fig. S1B). However, there were no significant differences in the concentrations of indole GSLs (GBS, 1MeGBS, 4MeGBS) (Fig. S1B). These findings suggest that the selection process aimed at reducing seed GC simultaneously targeted aliphatic and aromatic GSLs in the leaves, resulting in a substantial reduction in their content, while indole GSLs remained unaffected.

### Selective-sweep analysis unveils the genetic footprints left by intensive low-GC selection

**Fig. 3:**
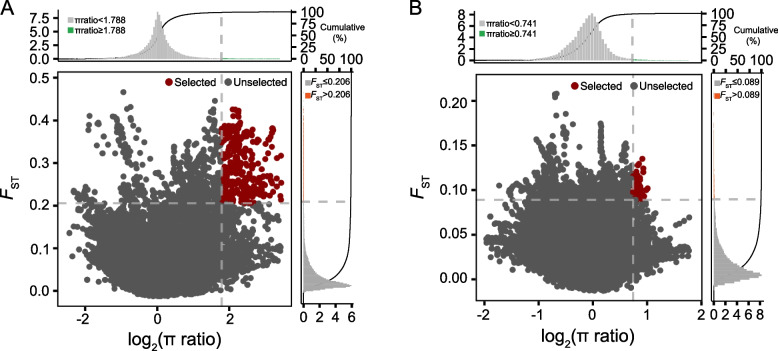
Candidate intervals based on π and F_ST_ between different GSL content in seeds and leaves. **A** Selective-sweep analysis of different accessions with GSL content above 90 μmol/g and below 30 μmol/g in seeds. Selection signals were screened based on log2(π ratio) and _F_ST, with red dots representing the top 1% of selection signals. **B** Selective-sweep analysis of different accessions with GSL content above 40 μmol/g and below 20 μmol/g in leaves. Selection signals were screened based on log2(π ratio) and _F_ST, with red dots representing the top 1% of selection signals

To investigate the distinct selection signals associated with low GC in seeds, we classified germplasm accessions with GC above 90 μmol/g as the H-GC-S type and those below 30 μmol/g as the L-GC-S type. We then conducted selective-sweep analyses between the H-GC-S and L-GC-S types. The selection intervals were defined as the top 1% based on log2(π ratio) (nucleotide diversity) and *FST* values. Through this approach, we identified a total of 29,508 SNPs (single nucleotide polymorphisms) within 625 candidate genes associated with selection signals for GC in seeds (Fig. 3A; Table S3). Although breeding for 'double low' traits primarily targeted GC in seeds, the genes involved in GSL synthesis in seeds also influence GSL levels in leaves. We extended our analysis to leaves, defining high leaf GC accessions (H-GC-L) as those with GC content above 40 μmol/g and low leaf GC accessions (L-GC-L) as those below 20 μmol/g. Selective-sweep analyses for leaves revealed 11,522 SNPs in 193 genes (Fig. 3B; Table S4). This suggests that the selection for low GC seeds impacted a considerable proportion of genes in the genomes of L-GC-L accessions.

The selection for the L-GC-S type resulted in allelic changes in several genes likely related to disease resistance, including orthologues of AT5G11250, BURNOUT1 (BNT1), which are involved in stress responses (BnaC03G0717600ZS, BnaC03G0717700ZS, BnaC03G0727600ZS, BnaC03G0727800ZS, BnaC03G0727900ZS), *AT3G44480 (RECOGNITION OF PERONOSPORA PARASITICA* 1, RPP1) (BnaC03G0727400ZS, BnaC03G0727700ZS), AT4G19500 (RPP2A) (BnaC03G0712400ZS, BnaC03G0712500ZS), *AT4G19510* (RPP2B) (BnaC03G0711800ZS, BnaC03G0712100ZS, BnaC03G0712600ZS), and AT3G04220 (NLR, nucleotide-binding domain leucine-rich repeat receptors) (BnaC03G0727500ZS), which are responsible for plant immunity and resistance to fungal pathogens (Table S3). Notably, selection for low GC left signatures on 3,477 SNPs corresponding to 80 genes common to both seeds and leaves (Table S5). These genes were identified within three contiguous sliding windows on chromosome C09, spanning 3,720,001–3,950,000, 4,230,001–4,390,000, and 4,640,001–4,800,000. Among these genes, *BnaC09.MYB28* (BnaC09G0066500ZS), a known transcription factor (TF) crucial for the aliphatic GSL synthesis pathway, was prominently featured in both organs (Hirai et al. 2007). We speculate that these specific chromosomal regions have experienced strong selection pressure during 'double low' breeding, leading to the fixation of alleles associated with genes that likely play a direct role in the synthesis or regulation of GSLs. This, in turn, affects GC in both leaves and seeds. The identification of *BnaC09.MYB28* highlights its critical role in GSL synthesis across various plant tissues.

### Genome-wide association studies reveal candidate genes associated with GC variations in seeds and leaves

**Fig. 4:**
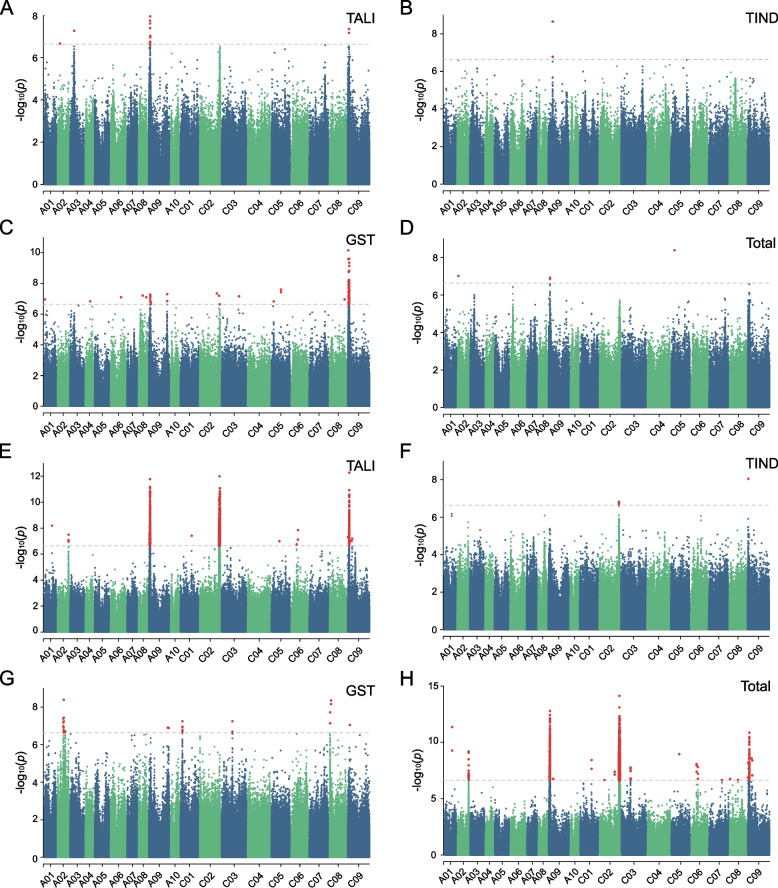
Genome-wide association study on GC in leaves and seeds. **A**-**D** Manhattan plots of TALI (**A**), TIND (**B**), GST (**C**), and total GSL content (GC; **D**) in leaves. (**E**–**H**) Manhattan plots of TALI (**E**), TIND (**F**), GST (**G**), and total GC (**H**) in seeds. Abbreviations for specific GSL species and their corresponding full names are: GST (Gluconasturtiin), TALI (Total aliphatic GSL), TIND (Total indole GSL), Total (Total GC)

In order to identify candidate genes associated with GC variation in leaves and seeds, GWASs were conducted on total GC and various GSL categories (Fig. 4; Figs. S3 and S4). The SNPs showing significant associations (-log_10_(*p*) > 6.63) with GC variations in leaves and seeds were compiled in Tables S6 and S7, respectively. A total of 303 genes were found to be associated with the variation in various GSL categories and/or individual species in leaves, while 1,015 genes were associated with similar variations in seeds. Specifically, in leaves, 61, 6, 197, and 23 genes were associated with levels of aliphatic, indole, aromatic, and total GC, respectively. Conversely, in seeds, 627, 11, 57, and 906 genes were associated with variations in aliphatic, indole, aromatic, and total GC, respectively (Tables S8, S9). Among the identified genes, 56 were associated with both aliphatic and total GC variations in both seeds and leaves. However, there were no overlapping genes between the two organs associated with variations in indole and aromatic GC (Fig. 7A; Tables S8 and S9). Notably, among the 56 genes related to aliphatic GC shared between leaves and seeds, several putative TFs were identified, including orthologs of the *Arabidopsis* F-box protein (*BnaA09G0061700ZS*), NAC domain protein *AT5G24950* (*BnaA09G0067200ZS*), a pseudo-response regulator (*BnaA09G0067500ZS*), Transcription factor TFIIIB component (*BnaA09G0070900ZS*), and TCP DOMAIN PROTEIN 7 (*BnaA09G0073000ZS*).

### Transcriptome analysis reveals differentially expressed genes between low- and high-GC genotypes in leaves and seeds

**Fig. 5:**
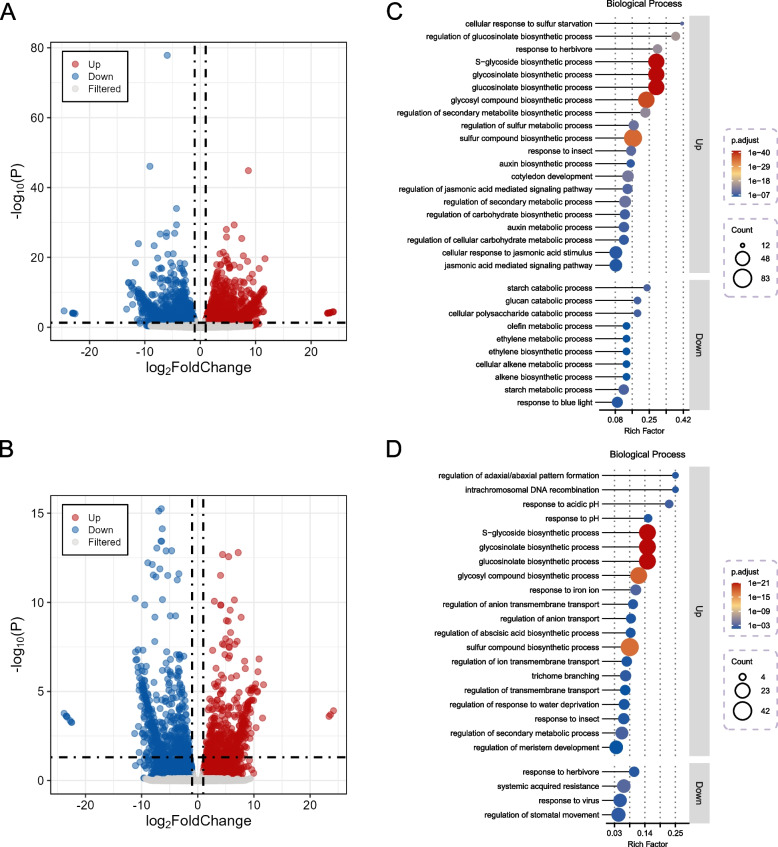
Identification and GO enrichment analysis of differentially expressed genes (DEGs) in leaves and seeds. **A**, **B** Volcano plots illustrating the DEGs identified in leaves (A) and seeds (B). The significance thresholds for DEGs were set at |log_2_(Fold Change)|> 1 and an *p* value < 0.05. Up-regulated genes (URGs) are depicted in red, down-regulated genes (DRGs) in blue, and genes with no significant changes are shown in gray. **C**, **D**) Gene Ontology (GO) enrichment analysis of DEGs identified in leaves (C) and seeds (D). The figures display the top 20 most significantly enriched GO terms for URGs and the top 10 most significantly enriched GO terms for DRGs

To gain deeper insights into the mechanisms underlying GSL synthesis in leaves, we selected two H-GC-L accessions (R4222 and R4845) and two L-GC-L accessions (R4634 and R4897) for RNA-seq analysis. A comparative analysis between H-GC-L and L-GC-L revealed a total of 7,694 differentially expressed genes (DEGs) (|log2(Fold Change)|> 1, *p* value < 0.05) (Fig. 5A; Table S10). Specifically, we identified 4,035 up-regulated genes (URGs) and 3,659 down-regulated genes (DRGs). To investigate the most significant biological processes associated with these DEGs, we performed Gene Ontology (GO) enrichment analysis. The top three biological processes based on the enrichment factor included cellular response to sulfur starvation, regulation of GSL biosynthetic and response to herbivore (Fig. 5C). Concurrently, we conducted DEG analysis using two H-GC-S accessions (R4222 and R4950) and two L-GC-S accessions (R4775 and R4434) for RNA-seq. Comparing H-GC-S to L-GC-S, we identified a total of 4,154 DEGs (|log2(Fold Change) |> < 0.05) (Fig. 5A; Table S10). Specifically, we identified 4,035 up-regulated genes (URGs) and 3,659 down-regulated genes (DRGs). To investigate the most significant biological processes associated with these DEGs, we performed Gene Ontology (GO) enrichment analysis. The top three biological processes based on the enrichment factor included cellular response to sulfur starvation, regulation of GSL biosynthesis, and response to herbivory (Fig. 5C). Concurrently, we conducted DEG analysis using two H-GC-S accessions (R4222 and R4950) and two L-GC-S accessions (R4775 and R4434) for RNA-seq. Comparing H-GC-S to L-GC-S, we identified a total of 4,154 DEGs (|log2(Fold Change)|> 1, *p* value < 0.05), including 2,080 URGs and 2,074 DRGs (Fig. 5B; Table S11). Based on the enrichment factor, the top three biological processes associated with these DEGs were regulation of adaxial/abaxial pattern formation, intrachromosomal DNA recombination and response to acidic pH (Fig. 5D). Notably, the biological processes such as S-glycoside biosynthesis, glycosinolate biosynthesis, GSL biosynthesis, glycosyl compound biosynthesis, and sulfur compound biosynthesis were most significantly enriched in both the DEGs between H-GC-L and L-GC-L and the DEGs between H-GC-S and L-GC-S (Fig. 5 C, D).

**Fig. 6:**
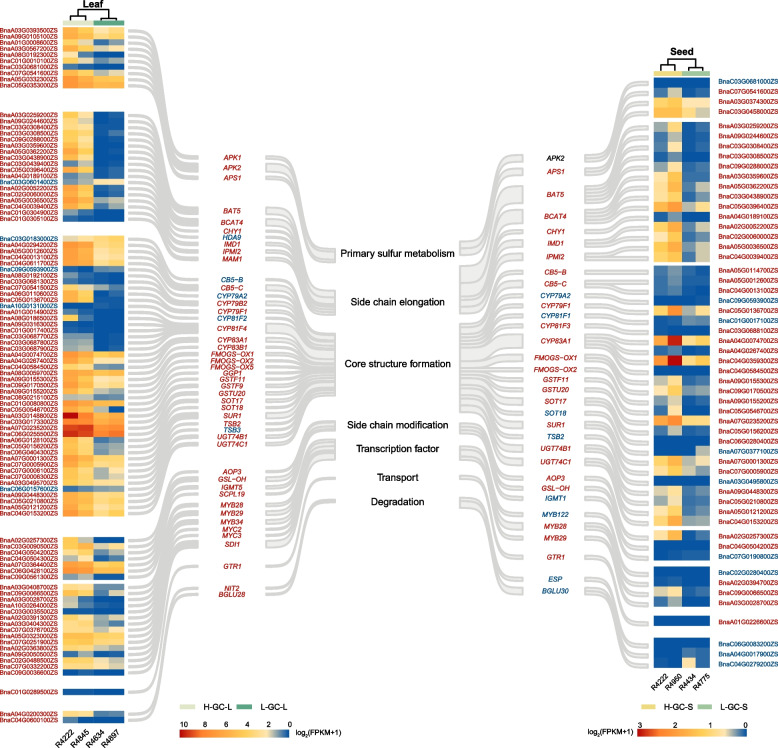
DEGs in the glucosinolate biosynthesis pathway that differentiate H-GC and L-GC types in leaves and seeds. The DEGs in leaves are displayed on the left side, while the DEGs in seeds are shown on the right. URGs are indicated in red, while DRGs are represented in blue. Genes marked in black signify differences in expression patterns between their homologous copies. H-GC-L refers to high GC in leaves, L-GC-L refers to low GC in leaves, H-GC-S refers to high GC in seeds, and L-GC-S refers to low GC in seeds

Among the 7,694 DEGs identified between H-GC-L and L-GC-L accessions, 99 had *Arabidopsis* orthologues known for their involvement in GSL synthesis. Of these, 94 were URGs and 5 were DRGs in the H-GC leaves. Specifically, 10, 18, 46, 7, 15, 1, and 2 DEGs corresponded to 3, 7, 23, 4, 6, 1, and 2 *Arabidopsis* orthologues recognized for their roles in primary sulfur metabolism, side chain elongation, GSL core molecular structure formation, side chain modification, transcription factor regulation, transport, and GSL degradation, respectively (Fig. 6). Similarly, among the 4,154 DEGs identified between H-GC and L-GC seeds, 55 had *Arabidopsis* orthologues associated with GSL synthesis. Within these 55 DEGs, 45 were URGs and 10 were DRGs in the H-GC seeds. Specifically, 4, 14, 26, 3, 4, 1, and 3 DEGs corresponded to 2, 5, 17, 3, 3, 1, and 2 *Arabidopsis* orthologues involved in primary sulfur metabolism, side chain elongation, GSL core molecular structure formation, side chain modification, transcription factor regulation, transport, and GSL degradation, respectively (Fig. 6). Interestingly, genes orthologous to *Arabidopsis PROTEIN KINASE 2B (APK2)* and *TRYPTOPHAN SYNTHASE BETA TYPE 2 (TSB2)* were upregulated in H-GC leaves relative to L-GC leaves (Fig. 6, left), but downregulated in H-GC seeds relative to L-GC seeds (Fig. 6, right).

### *BnaC09.MYB28* plays a critical role in regulating GST synthesis in leaves

**Fig. 7:**
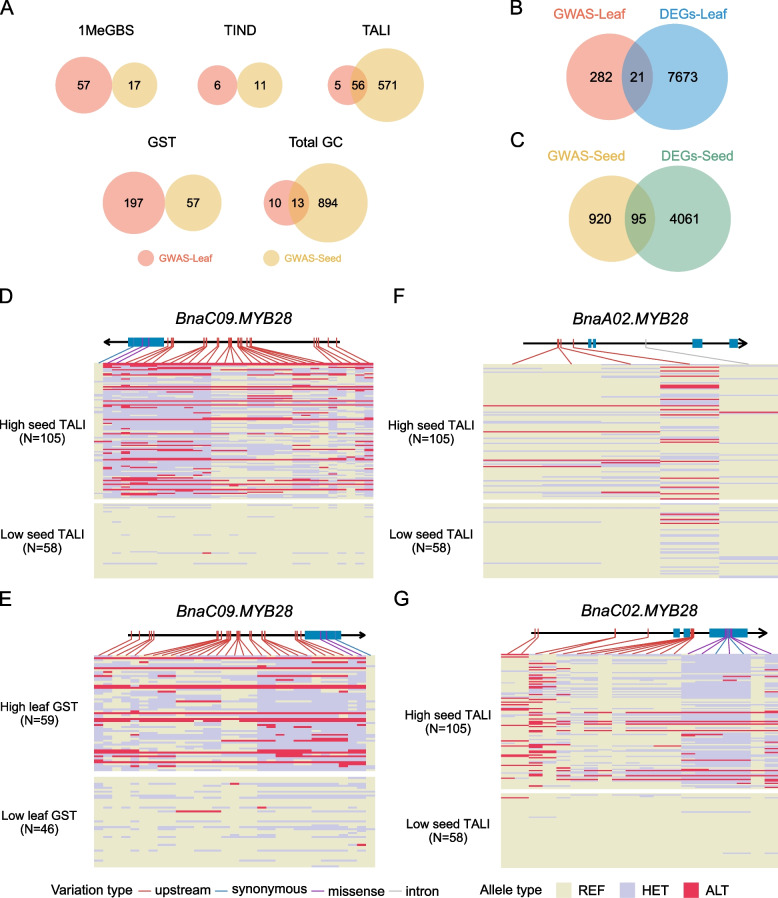
Identification of candidate genes for genome-wide association study (GWAS) of glucosinolates content in leaves and seeds. **A** Cross-analysis of candidate genes associated with the variation of various GSL categories and/or individual species identified by GWAS in leaves and seeds. **B**, **C** Cross-analysis of candidate genes involved in GSL synthesis based on GWAS and RNA-seq in leaves (B) and seeds (C). (D-G) A sketch showing the gene structure and allelic changes in the gene and the upstream 3 kb putative promoter region of *BnaC09.MYB28* (**D**), *BnaA02.MYB28* (**F**), and *BnaC02.MYB28* (**G**) between germplasms with high and low TALI content in seeds, as well as *BnaC09.MYB28* (**E**) among accessions with high and low GST content in leaves. The yellow, blue, and red colors indicate SNPs homozygous for the reference genotype (REF), heterozygous SNPs (HET), and SNPs homozygous for the non-reference allele (ALT), respectively. Abbreviations for specific GSL species and their corresponding full names are as follows: GST (Gluconasturtiin), TALI (Total aliphatic GSL), TIND (Total indole GSL), Total (Total GSL content)

To identify the key regulators driving the mechanisms of GSL synthesis in leaves and seeds, we conducted a comprehensive analysis by integrating the results of GWAS and DEG analysis. In the context of leaves, we discovered 21 genes that not only exhibited a significant association with GC variation but also demonstrated substantial disparities in transcription levels between the accessions with high- and low-GC leaves (Fig. 7B; Table S12). Among these genes, one particular locus, *BnaC09.MYB28*, possessed an orthologous gene, *AtMYB28*, in *Arabidopsis*, which has previously been reported to regulate GSL biosynthesis. Similarly, we identified 95 genes in seeds through the cross-analysis of GWAS and DEGs, encompassing not only *BnaC09.MYB28* but also *BnaA02.MYB28* (*BnaA02G0394700ZS*) (Fig. 7C; Table 2; Table S12). Upon comparing the regulators of GSL biosynthesis in leaves and seeds, we observed that *BnaC09.MYB28* was the sole *BnMYB28* gene responsible for determining low- or high-GC leaves. Conversely, two homologous *BnMYB28* genes were involved in regulating GSL biosynthesis in seeds (Table 2). Recognizing the potential significance of *MYB28* in governing GSL synthesis in rapeseed, we further examined the divergence of SNPs within the coding sequence and the 5'-regulatory region located 3 kb upstream of the coding sequence of the different *MYB28* copies identified. Notably, the SNPs within the coding sequences and 5'-regulatory regions of *BnaC09.MYB28* displayed significant differentiation between germplasms with high and low TALI content in seeds, as well as between those with high and low GST content in leaves (Fig. 7D, E). Similar SNP differentiation was also observed in *BnaA02.MYB28* and *BnaC02.MYB28* (*BnaC02G0527500ZS*), two *MYB28* copies associated with different TALI content in seeds (Fig. 7F, G). In the majority of H-GC accessions, alternative or heterozygous alleles were prevalent compared to the reference genome of ZS11, a ‘double low’ Chinese rapeseed cultivar. In contrast, most L-GC accessions exhibited a substantial number of homozygous alleles identical to those found in the ZS11 reference genome. In addition to *BnMYB28*, our cross-analysis also identified other TFs potentially involved in the regulation of GSL synthesis, specifically the orthologues to *PSEUDO-RESPONSE REGULATOR 5* (*APRR5*), *APRR1*, *TFIIIB*, *MYB86*, and *bHLH99* in *Arabidopsis* (Table 2). Remarkably, these TFs were found to play a role in either leaves or seeds, contributing to the complex regulation of GSL biosynthesis in different rapeseed tissues.

**Table 2:** Candidate genes identified by cross-analysis of GWAS and RNA-seq analysis

Bna_Gene_ID	Organ	*Arabidopsis* orthologue	Annotation
*BnaA02G0394700ZS*	Seed	*AT5G61420*/*MYB28*	Transcription factor MYB28
*BnaA03G0293800ZS*	Seed	*AT3G04290*/*LTL1*	GDSL esterase/lipase LTL1
*BnaA09G0028200ZS*	Seed	*AT4G02740*	F-box protein SKIP17
*BnaA09G0030000ZS*	Seed	*AT4G03030*	F-box/kelch-repeat protein OR23
*BnaA09G0050300ZS*	Seed	*AT5G48810*/*CYTB5-D*	Cytochrome B5
*BnaA09G0056000ZS*	Seed	*AT5G27380*/*GSH2*	GLUTATHIONE SYNTHETASE 2
*BnaA09G0067500ZS*	Leaf	*AT5G24470*/*APRR5*	Two-component response regulator-like APRR5
*BnaA09G0070900ZS*	Seed	*AT4G39160*	Transcription factor TFIIIB component B
*BnaC02G0506000ZS*	Seed	*AT5G26660*/*MYB86*	Transcription factor MYB86
*BnaC02G0515900ZS*	Seed	*AT1G59620*	Probable disease resistance protein
*BnaC02G0531700ZS*	Seed	*AT5G62310*/*IRE*	Probable serine/threonine protein kinase IRE
*BnaC02G0548500ZS*	Seed	*AT5G65320*/*bHLH99*	Transcription factor Bhlh99
*BnaC09G0033100ZS*	Seed	*AT3G28330*	F-box protein
*BnaC09G0036600ZS*	Leaf	*AT5G48850*/*SDI1*	Protein SULFUR DEFICIENCY-INDUCED 1
*BnaC09G0055400ZS*	Seed	*AT5G25120*/*CYP71B11*	Cytochrome P450 71B11
*BnaC09G0056400ZS*	Seed	*AT4G39160*	Transcription factor TFIIIB component B
*BnaC09G0066100ZS*	Leaf	*AT5G61380*/*APRR1*	Two-component response regulator-like APRR1
*BnaC09G0066500ZS*	Leaf, seed	*AT5G61420*/*MYB28*	Transcription factor MYB28
*BnaC09G0170500ZS*	Seed	*AT1G65860*/*FMO GS-OX1*	Flavin-containing monooxygenase FMO GS-OX1

## Discussion

The reduced glucosinolate content (GC) in rapeseed cake has significantly enhanced its feed value, prompting a shift in some countries, such as China, from treating the cake as organic fertilizer to utilizing it as high-quality protein feed. It is widely accepted—and our own research findings corroborate—that a robust positive correlation exists between GC in leaves and seeds (Fig. 2; Liu et al. 2020b). Consequently, while breeders have successfully decreased the overall GC in seeds, this has inevitably resulted in a concurrent reduction in GC in other vegetative organs, including leaves. A series of studies have consistently demonstrated that leaf glucosinolates (GSLs) contribute to resistance against pests, diseases, and bird damage (Zhao et al. 2016; Liu et al. 2021; Qin et al. 2023). Additionally, leaf GSLs possess significant nutritional value for human health (Becker and Juvik 2016). In recent years, rapeseed leaves have been harvested as a vegetable in certain regions, augmenting the crop's overall economic value. Maintaining substantial GC in leaves not only enhances the adaptability of rapeseed to its environment but also increases its overall value. Thus, achieving a delicate balance between maintaining a relatively low GC in seeds while maximizing the GC in vegetative organs—particularly leaves—represents a crucial task for breeders. To accomplish this objective, a detailed understanding of the distinct mechanisms governing GSL biosynthesis in rapeseed seeds and leaves is imperative. Currently, all ‘double low’ varieties can trace their low-GC genes back to the Polish low-GC-S cultivar *'Bronowski'*. However, due to the reliance on monotonous parental sources, breeding of low-GC-S varieties has resulted in narrowed genetic diversity in rapeseed.

Our SSA has revealed genetic imprints within the rapeseed genome, attributable to decades of intensive breeding for low-GC seeds. These imprints indicate the fixed linkage of specific alleles of certain genes. On chromosome C09, we identified three continuous sliding windows in both L-GC-S and L-GC-L accessions, suggesting that these regions, which consist of 48 associated genes, are under strong selection pressure. Notably, *MYB28* (also known as *HIGH ALIPHATIC GLUCOSINOLATE 1*, *HAG1*) has been previously reported to regulate the synthesis of aliphatic GSLs (Hirai et al. 2007). The expression of *MYB28* is significantly induced by glucose, suggesting a transcription factor (TF) mechanism that integrates carbohydrate availability in response to biotic challenges (Gigolashvili et al. 2007). The identification of *BnaC09.MYB28* underscores its critical role in GSL synthesis in both leaves and seeds. However, the impact of another TF, WRKY30, on GSL synthesis has not been extensively studied. Several WRKY family TFs are known to regulate indole GSL synthesis (Schön et al. 2013; Tao et al. 2022). WRKY TFs also respond to pathogens, elicitors, and defense signals related to phytohormones (Chen et al. 2019). Moreover, they play pivotal roles in plant responses to abiotic stresses, including wounding, drought, salinity, heat, and cold stresses (Chen et al. 2012). Therefore, WRKY30 might control the expression of stress-related genes, coordinate signaling pathways, and enhance plant resilience to both biotic and abiotic stresses, promoting survival and adaptation in challenging environments. The selection pressure on *MYB28* and *WRKY30* could potentially reduce the adaptability of L-GC-S varieties to stressful environments. In addition to *MYB28* and *WRKY30*, several disease-related genes, including orthologues to *Arabidopsis BNT1*, *RPP1*, *RPP2A*, *RPP2B*, and *NLR*, were also identified. *BNT1* is reported to encode a Toll/Interleukin1 receptor-nucleotide binding site leucine-rich repeat protein and is a key gene in response to environmental stresses in plants (Sarazin et al. 2015). *RPP1*, *RPP2A*, and *RPP2B* mediate disease resistance to the oomycete pathogen *Peronospora parasitica* and rose powdery mildew (*Podosphaera pannosa*) (Linde et al. 2004; Sinapidou et al. 2004). Additionally, *NLRs* play a pivotal role in plant immunity by integrating signals from both pathogen-associated molecular pattern-triggered immunity (PTI) and effector-triggered immunity (ETI) pathways to activate defense responses (Lang et al. 2022). During the selection process for the L-GC-S genotype, some unfavorable disease resistance alleles appear to be fixed. Overall, these TFs, disease-related genes, and others listed in Tables S3 and S4 represent valuable targets for efforts aimed at diversifying the genetic foundation of L-GC-S cultivars.

To breed genotypes with low-GC seeds while maintaining higher GC in leaves, it is essential to disrupt the strong positive correlation observed between GC in seeds and leaves. Although our results demonstrate a significant positive correlation in total GC between leaves and seeds, further analysis of individual GSL components reveals that this correlation is primarily driven by aliphatic and aromatic GSLs (Fig. 2). Notably, there is no significant correlation in indole GC between leaves and seeds (Fig. 2D). This suggests that by gaining a deeper understanding of the distinct genes governing indole GSL synthesis in leaves and seeds, we can selectively manipulate the GC in a specific tissue by targeting specific genes.

Our GWAS on GC variations in leaves and seeds reveals that the synthesis of GSL components involves both shared and unique genes between the two tissues. We identified a total of 61 genes associated with aliphatic GC in leaves, of which 56 genes (91.8%) were also associated with aliphatic GC in seeds. However, these 56 genes represent only 8.9% of the 627 genes associated with aliphatic GC in seeds (Fig. 7A; Tables S8 and S9), suggesting that selecting or manipulating the remaining 571 genes (91.1%) would be unlikely to change the aliphatic GC in leaves but only in seeds. In contrast, we did not find any genes shared between leaves and seeds for indole GC. Overall, the number of genes involved in GSL synthesis in seeds is significantly higher than in leaves (Fig. 7A; Tables S8 and S9). For instance, there were 627 and 11 genes associated with aliphatic and indole GC in seeds, respectively, while in leaves, there were only 61 and 6 genes associated with these respective GSL categories. However, there were more genes associated with aromatic GC in leaves (197) compared to seeds (57). The difference in the number of genes involved in GSL synthesis between seeds and leaves could be attributed to tissue-specific regulation, developmental requirements, functional diversity, and metabolic specialization of the tissues.

We conducted DEG analyses between accessions with low- and high-GC leaves, as well as between accessions with low- and high-GC seeds (Fig. 5 A, B; Tables S10 and S11). In the comparison of L-GC-S and H-GC-S accessions, as well as L-GC-L and H-GC-L accessions, we identified 99 and 55 genes associated with GSL synthesis, regulation, and degradation in the DEGs of seeds and leaves, respectively. Notably, *MYB28* and *MYB29* play critical roles as key components of the regulatory network governing aliphatic GSLs, while *branched-chain aminotransferase 4* (*BCAT4*) and *bile acid transporter 5* (*BAT5*) are implicated in the elongation of side chains in aliphatic and aromatic GSLs. Furthermore, *FMO GS-OXs* are involved in the modification of side chains in aliphatic GSLs (Fig. 6; Tables S10 and S11; Schuster et al. 2006; Hansen et al. 2007; Hirai et al. 2007; Gigolashvili et al. 2009).

These findings indicate a significant correlation between aliphatic and aromatic GSLs in both leaves and seeds, linked to consistent gene expression patterns in these tissues. The gene *GSL-OH*, which encodes a 2-oxoacid-dependent dioxygenase, is responsible for converting GBN to GNL and exclusively for converting GNA to PRO in Met-derived aliphatic GSLs (Hansen et al. 2008; Qin et al. 2023). Our study identified three copies of *BnGSL-OH* that were differentially expressed between H-GC-L and L-GC-L accessions, but only one copy was identified as a DEG between H-GC-S and L-GC-S accessions. This may partially explain why PRO is detected in leaves.

Regarding the regulation of indole GSLs, we detected three copies of *BnMYB34* in leaves and only one copy of *BnMYB122* in seeds. MYB34 and MYB122 are recognized as crucial positive regulatory TFs for indole GSLs (Gigolashvili et al. 2007; Frerigmann and Gigolashvili 2014). Consequently, we conclude that the distinct expression patterns of *BnMYB34* and *BnMYB122* in leaves and seeds may result in variations in the synthesis and metabolism of indole GSLs, leading to the lack of correlation in the levels of indole GSLs between the two tissues. Some members of cytochrome P450 monooxygenases, such as CYP81F1-3, can catalyze hydroxylation reactions of the GSL indole ring, leading from GBS to OHGBS (Pfalz et al. 2011). The differing numbers of these DEGs between H- and L-GC in leaves and seeds might partly explain the identification of OHGBS only in seeds.

To identify the key genes responsible for the divergence in GSL synthesis mechanisms between leaves and seeds, we conducted a comprehensive analysis by integrating the results of GWAS and DEG analysis. Our investigation revealed 21 genes significantly associated with GC variation in leaves and 95 genes in seeds, both displaying substantial differences in transcription levels between high- and low-GC accessions (Fig. 7 B, C; Table 2; Table S12). Upon comparing the regulators of GSL biosynthesis in leaves and seeds, we observed that *BnaC09.MYB28* was the sole *BnMYB28* gene determining low- or high-GC leaves. In contrast, three *BnMYB28* homologues—namely, *BnaC09.MYB28*, *BnaA02.MYB28*, and *BnaC02.MYB28*—were involved in regulating GSL biosynthesis in seeds. This suggests that to manipulate GC in seeds by targeting *MYB28*, it would be more appropriate to focus on the *MYB28* genes located on chromosomes A02 and C02, as manipulating these two *BnMYB28* genes is unlikely to lead to a concurrent decrease in GC in leaves. This conclusion requires further molecular experiments to verify whether this strategy can achieve a genotype characterized by low seed GSLs but high leaf GSLs. In addition to the aforementioned *BnMYB28* genes, other *BnMYB28* homologs are present on chromosomes C07, A03, and A09 in the genomes of varieties such as Zheyou7, Darmor-bzh, Janetzkis (123,456 Schlesischer), and Lorenz (Schilbert et al. 2022). However, their association with GC was not detected in our study due to our use of the ZS11 genome as the reference for SNP mapping. A limitation of this study is the failure to utilize a widely accepted pan-genome reference for *Brassica napus*, which is not yet available for this species.

In addition to *BnaC09.MYB28*, other transcription factors (TFs) that may be simultaneously involved in glucosinolate (GSL) synthesis in seeds or leaves include *BnaA09G0067500ZS*, *BnaC09G0066100ZS*, *BnaC09G0056400ZS*, *BnaC02G0548500ZS*, and *BnaC02G0506000ZS* (Table 2). Among these, *BnaA09G0067500ZS* encodes *Arabidopsis Pseudo-Response Regulator 5* (*APRR5*), and *BnaC09G0066100ZS* encodes *APRR1*, both of which are part of the *Arabidopsis* circadian clock system (Matsushika et al. 2000). This system plays a crucial role in coordinating plant physiological processes, including flowering and the production of secondary metabolites (Yamamoto et al. 2003). *APRR5* may modulate the expression of genes involved in GSL biosynthesis based on the circadian rhythm, thereby affecting their accumulation in plants. It is possible that *APRR5* or *APRR2* interacts with other regulatory proteins or TFs to form complexes that regulate GSL biosynthesis. Additionally, *BnaA09G0070900ZS* and *BnaC09G0056400ZS* are orthologues of *Arabidopsis* TFIIIB, which may interact with other regulatory proteins involved in GSL biosynthesis, forming complexes that enhance or inhibit the activity of biosynthetic enzymes, subsequently influencing GSL production. Furthermore, the TFIIIB complex, which contains TFIIIB domains, is known to participate in chromatin remodeling, altering the accessibility of genes involved in GSL biosynthesis (Wang and Roeder 1995; Gelev et al. 2014). This remodeling can impact the binding of other regulatory elements to the DNA, ultimately influencing gene expression. *BnaC02G0548500ZS* and *BnaC02G0506000ZS*, homologues of bHLH and *MYB86*, respectively, may jointly act with other MYB and bHLH TFs to regulate GSL synthesis (Schweizer et al. 2013; Frerigmann et al. 2014).

Genome-Wide Association Studies (GWAS) are a powerful tool for identifying genetic variations associated with important agronomic traits. One significant advantage of GWAS is its ability to detect genetic variations without prior knowledge of gene function, allowing for an unbiased approach (Pearson and Manolio 2008). This approach facilitates the discovery of new genetic associations, including rare or novel variants that may significantly affect traits. In our previous studies, GWAS has played a pivotal role in identifying genes that regulate various traits in rapeseed, such as flowering time (Wu et al. 2019; Xu et al. 2023), leaf trichome density (Xuan et al. 2020), seed oil content (Wang et al. 2020), drought tolerance (Zhu et al. 2020), tocopherol content and composition (Huang et al. 2023), leaf wax thickness (Long et al. 2023), shade tolerance (Li et al. 2023), and petal size (Wang et al. 2023). The advantages of our GWAS population include its manageable size for field experiments with repetitions, while still retaining a large number of SNPs, which were curated through the resequencing of 991 germplasm accessions originating from 38 countries/regions worldwide (Wu et al. 2019).

In summary, we found that the aliphatic GSL PRO was exclusively detected in leaves, while the indole GSL OHGBS was solely found in seeds. Aliphatic and aromatic GSLs, rather than indole GSLs, play a significant role in the positive correlation between GC in seeds and leaves. Therefore, selecting or manipulating for low indole GSLs, particularly OHGBS, in seeds is unlikely to reduce GSL levels in leaves. Our GWAS identified approximately 627 genes associated with variations in aliphatic GC in seeds. Manipulating 571 (91.2%) of these genes would likely have minimal impact on aliphatic GC in leaves. The gene *BnMYB28* plays a crucial role in regulating GC in both seeds and leaves. Manipulating *BnaC09.MYB28* would affect GC in both tissues. However, downregulating *BnaA02.MYB28* and/or *BnaC02.MYB28* would reduce GC in seeds without likely causing a concurrent reduction in GC in leaves.

## Materials and Methods

### Plant materials and genotyping

In this study, a core collection of 235 *Brassica napus* accessions was selected from a total of 991 germplasm accessions (Wu et al. 2019; Xuan et al. 2020). This subset was chosen for its manageable size, facilitating efficient field experiments with repetitions. Genotyping was performed by aligning 4,312,417 SNPs to the reference genome ZS11 v0 (https://yanglab.hzau.edu.cn/BnIR/germplasm_info?id=ZS11.v0). To improve data quality, genotype imputation was carried out using Beagle software, and non-biallelic markers were excluded with Bcftools. SNPs not meeting the threshold of a 5% minor allele frequency and having over 10% missing data were discarded. SNP annotations were systematically performed using snpEFF. The genotype data generated in this study is publicly available at https://github.com/YTLogos/BnaGWAS.

### Plant growth conditions and phenotyping

The materials for GC analysis were cultivated and collected from the experimental fields of the Jiaxing Academy of Agricultural Sciences in Jiaxing, China. The plants were grown in plots measuring 150 × 40 cm, with 8–12 plants per accession. Seeds that had naturally matured and air-dried were used for the GC determination. For leaf GC measurement, plants were grown in a plant growth room. Rapeseed seedlings were cultivated in seedling trays (8 × 4 cells, with individual cell dimensions of 58 mm × 20 mm × 110 mm). Growth room conditions were carefully controlled, with a light intensity of 12,000 lx, 68% relative humidity, and a photoperiod of 16 h of light at 23 °C and 8 h of darkness at 20 °C. The sixth true leaf from seedlings was harvested and freeze-dried for 48 h using a freeze dryer (FD-C12N, Jingfu, Shanghai, China).

The quantification of total and specific GSL components was performed using HPLC–DAD/UV. GSL extraction followed the methodology outlined by Maodzeka et al. (2019). Methanol was added to 10 mg of ground seed powder. For GSL isolation, a filter plate (Catalogue no. MAHVN4550, Millipore, Tempe, Arizona, U.S.A) was loaded with 30 mg of DEAE Sephadex A25, followed by sulfatase treatment and elution in 60% methanol and ddH2O using a vacuum manifold (WelVac 210, Rocker Scientific, New Taipei, Taiwan). Desulfo-glucosinolate separation and quantification utilized a Waters 1525 binary pump system, coupled with a column heater, 2707 series autosampler, and 2998 DAD detector (Waters Corporation, Milford, Massachusetts, U.S.A), all controlled by Empower 2 software. A Hypersil C18 column (5 µm particle size, 4.6 mm × 250 mm; Elite Analytical Instruments Co. Ltd, Dalian, China) was used, maintained at 30 °C. The injection volume was set at 45 µL, and detection occurred at 229 nm, employing water and acetonitrile as the mobile phase. Quantification relied on peak areas and published response factors, utilizing sinigrin as an internal standard (Brown et al. 2003), while identification of individual GSLs was achieved through HPLC-Electrospray Ionization-Mass Spectrometry (HPLC–ESI–MS) based on their distinctive m/z values (Olsen et al. 2016).

### Selective sweep analysis

Selective-sweep analysis (SSA) was performed to investigate genomic signatures across distinct group comparisons. Vcftools was used to calculate within-group π and between-group _F_ST. These calculations were conducted with a window size of 100 kb and a step size of 10 kb along each chromosome. The mean π and _F_ST values within each window were computed as population-level metrics for these parameters. Subsequently, the log2(π ratio) and _F_ST values were ranked in descending order, isolating the top 1% of windows. These windows were identified as regions exhibiting strong selection signals. Significant SNPs within these candidate regions were then extracted, and genes containing these SNPs within the 3 kb upstream region, the gene body (exons and introns), and the 500 bp downstream region were identified as being subject to selection.

### Determination of the population structure and genetic diversity

To enhance SNP selection based on linkage disequilibrium (LD), we filtered out non-linked SNPs using a window size of 50 kb, a step size of 10 SNPs, and a correlation threshold of 0.2. This filtering process yielded 380,022 high-confidence SNPs, which were subsequently used to infer population structure using Admixture. Population clusters (K values) were tested from 1 to 9, and fivefold cross-validation was employed to determine the optimal clustering scheme, identified by the lowest cross-validation error rate. Population structure was visualized using the Pophelper package in R. Additionally, principal component analysis (PCA) was performed with GCTA, using the first two principal components to represent genetic variation among the populations. SNP density across the genome was visualized using the CMplot package in R (Supplemental Fig. S2).

### Genome-wide association mapping of seed glucosinolate contents

GWASs were conducted using Efficient Mixed-Model Association eXpedited (EMMAX). The significance threshold for identifying high-quality trait-associated SNPs was determined by the formula *P* = 1/n (where n is the total number of SNPs; Long et al. 2023). SNPs that surpassed this threshold but appeared in isolation were discarded. The LD decay reaching r^2^ > 0.2 corresponded to a physical distance of 33.6 kb. Consequently, sequence regions spanning 33.6 kb adjacent to significantly associated SNPs were scrutinized for potential candidate genes.

### Transcriptome analysis

For the transcriptome analysis of leaves, raw sequence reads from the third true leaf at the seedling stage were downloaded from the NCBI Sequence Read Archive (SRA) under the project accession number PRJNA309367 (https://www.ncbi.nlm.nih.gov/sra/?term=PRJNA309367; Havlickova et al. 2018). For seed transcriptome analysis, seeds were harvested from siliques 20 days after flowering (DAF), immediately flash-frozen in liquid nitrogen, and stored at −80 °C for later analysis. RNA extraction, library construction, and paired-end sequencing were carried out using the Illumina sequencing platform (Illumina, San Diego, USA) at Personalbio Technology Corporation (Shanghai, China).

The quality of the sequencing data was initially assessed using FastQC. Adapter sequences, low-quality bases, and short reads were removed using Trimmomatic. The clean reads were then aligned to ZS11.v0 using HISAT2. Gene expression levels were quantified using featureCounts, while Cufflinks was employed to calculate expression values as fragments per kilobase of transcript per million mapped reads (FPKM). DEGs were identified using the DESeq2 package in R.

### Gene ontology enrichment analysis

The functional annotation data for our analysis was obtained from the EggNOG database, with the reference genome set as ZS11.v0. Protein sequences were annotated using EggNOG-mapper. An OrgDb package was constructed using the AnnotationForge package in R. Gene Ontology (GO) enrichment analysis of differentially expressed genes (DEGs) was performed using the OrgDb and clusterProfiler packages and visualized with the ggplot2 package in R.

## Supplementary Information

Supplementary Material 1

Supplementary Material 2

## Data Availability

The supporting data for Figures and Tables are available in Supplemental Figs. 1–6 and Supplemental Tables 1–12. The raw reads of the rapeseed accessions have been deposited in the public database of the National Center for Biotechnology Information under SRP155312 (https://www.ncbi.nlm.nih.gov/sra/SRP155312) and the China National Center for Bioinformation (NGDC) (https://ngdc.cncb.ac.cn/gsa/browse/CRA001854).

## Abbreviations

GC: Glucosinolates content
GSL: Glucosinolate
TALI: Total aliphatic GSLs
TIND: Total indole GSLs
TFs: Transcription factors
GWAS: Genome-wide association study
SSA: Selective-sweep analysis
SNPs: Single nucleotide polymorphisms
LD: Linkage disequilibrium
PCA: Principal component analysis
EMMAX: Efficient Mixed-Model Association eXpedited
DAF: Days after flowering
H-GC-S: High GC in seeds
L-GC-S: Low GC in seeds
H-GC-L: High GC in leaves
L-GC-L: Low GC in leaves
DEGs: Differentially expressed genes
URGs: Up-regulated genes
DRGs: Down-regulated genes
GO: Gene Ontology
