# Supplementary material for: Deciphering the heterogeneous glucosinolates composition in leaves and seeds: strategies for developing Brassica napus genotypes with low seed glucosinolates content but high leaf glucosinolates content
Source: Mol Hortic. 2025 May 1;5:23. doi: 10.1186/s43897-025-00147-1 (PMC12044725; doi:10.1186/s43897-025-00147-1)
Supplement: Supplementary file 1 — Supplementary Material 1 [file 43897_2025_147_MOESM1_ESM.pdf]

Supplementary information for

Deciphering the Heterogeneous Glucosinolates Composition in Leaves and Seeds: Strategies for Developing *Brassica napus* Genotypes with Low Seed Glucosinolates Content but High Leaf Glucosinolates Content

Mengxin Tu, Wenxuan Guan, Antony Maodzeka, Hongyu Zhou, Zi Zhang, Tao Yan, Shuijin Hua, Lixi Jiang

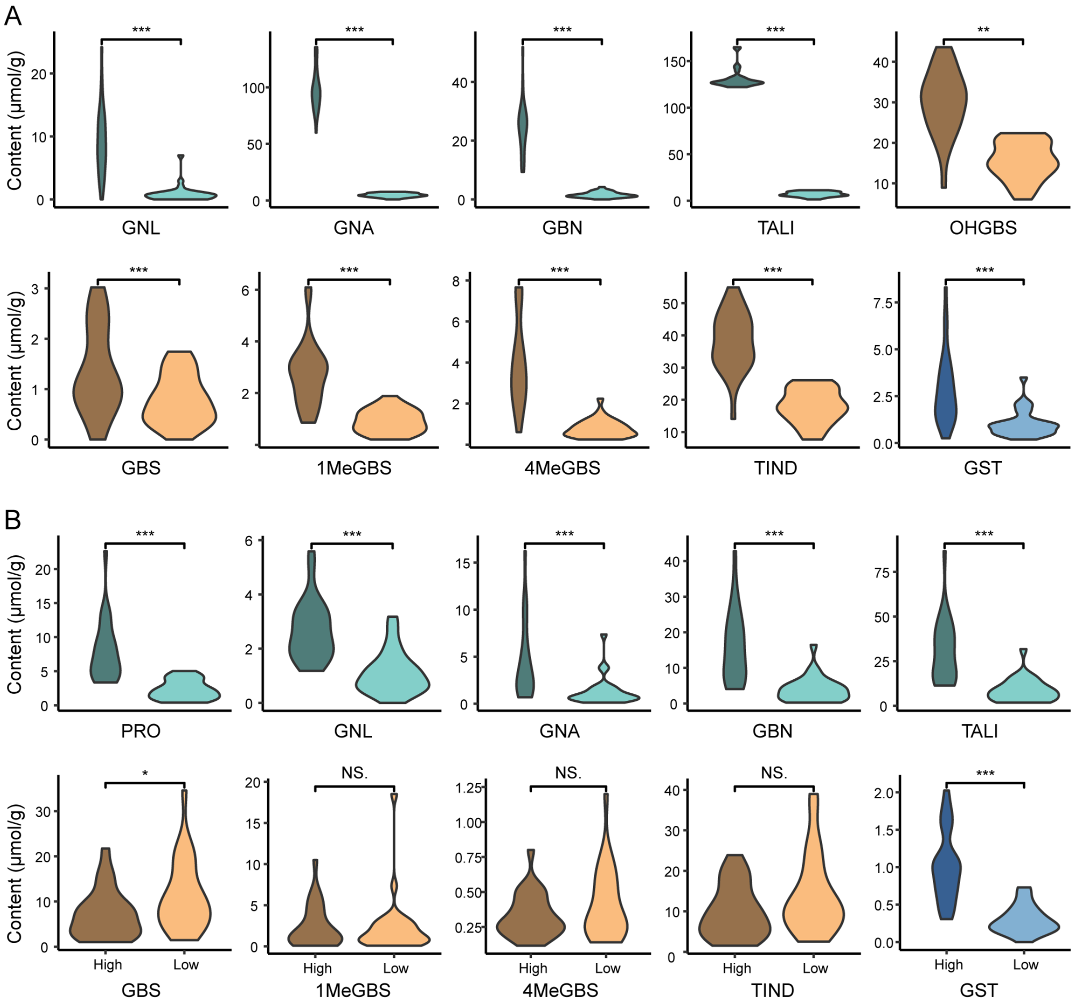

Supplementary Fig. S1. Comparison of GSL content between leaves and seeds among 60 selected *B. napus* accessions. (A-B) Violin plot showing the comparison of different component of GSLs in seeds (A) and leaves (B) in a subset of 60 materials with the highest seed GSLs content (GC) and lowest seed GC. Specific GSL abbreviations and their corresponding full names are as follows: PRO (Progoitrin), GNA (Gluconapin), GNL (Gluconapoleiferin), GBN (Glucobrassicanapin), GBS (Glucobrassicin), OHGBS (4-Hydroxyglucobrassicin), 4MeGBS (4-Methoxyglucobrassicin), 1MeGBS (1-Methoxyglucobrassicin), GST (Gluconasturtiin), TALI (Total aliphatic GSL), TIND (Total indole GSL).

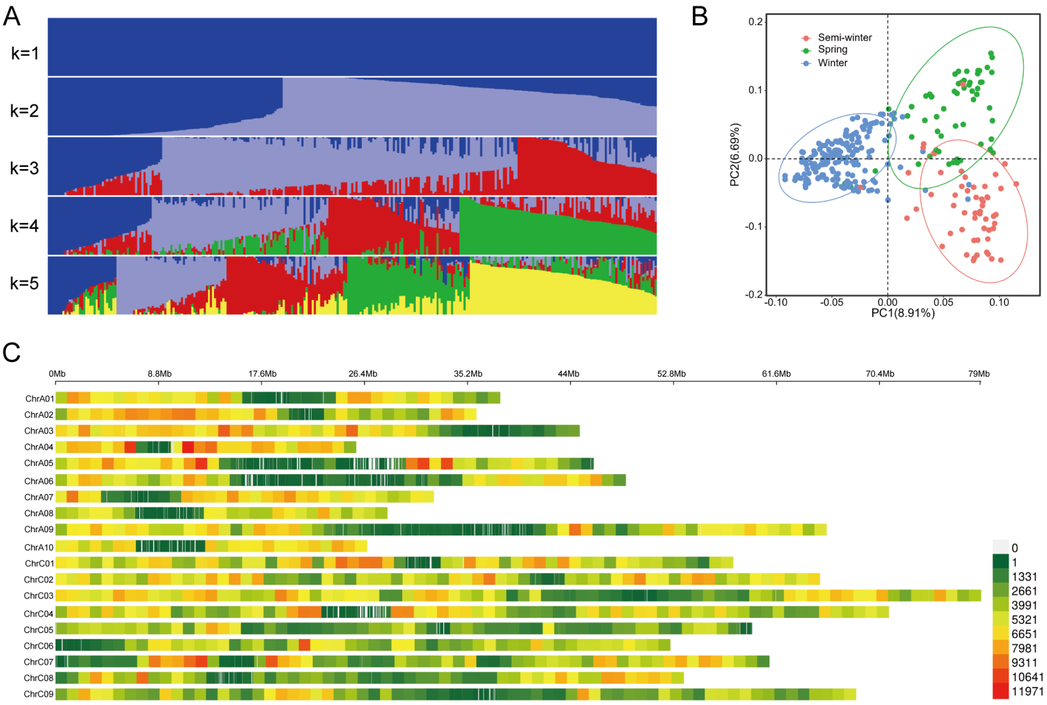

Supplementary Fig. S2. Population structure analysis of 235 core rapeseed germplasms. (A) Population structure of 235 rapeseed germplasm. The y axis quantifies cluster membership, while the x axis represents the different accessions. (B) Principal component analysis of the 235 germplasm. PC1, indicating 8.91% of the total variation, divides the winter type accessions from the spring type and semi-winter accessions, whereas PC2, representing 6.69% of the total variation, separates the semi-winter types from the spring types. Peach dots represent the semi-winter ecotype, green dots represent the spring ecotype, and blue dots represent the winter ecotype. (C) Single nucleotide polymorphism (SNP) density on the 19 chromosomes of 235 genomes. Different colors show various densities as displayed in the spectrum column.

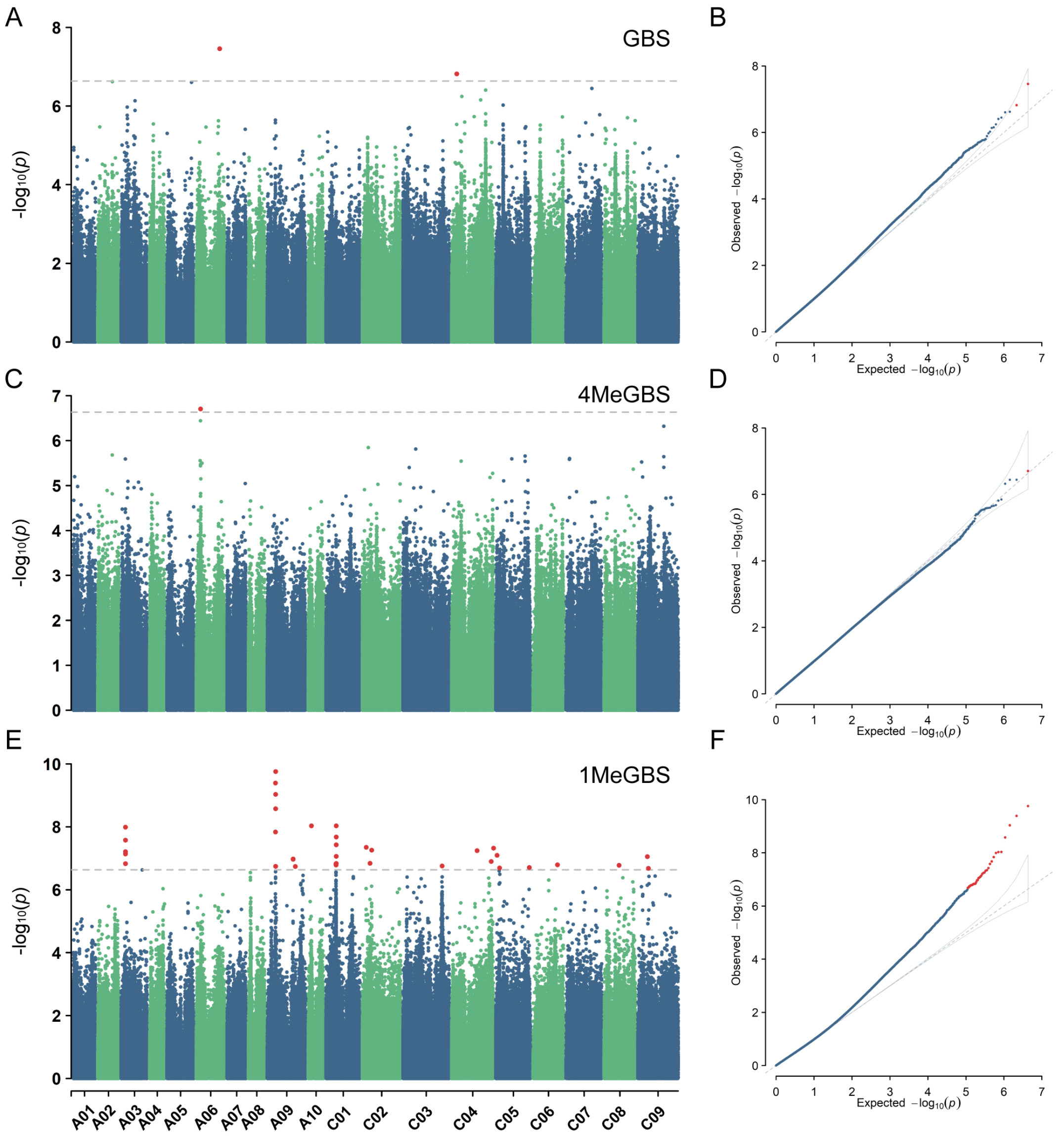

Supplementary Fig. S3. Genome-wide association study on indole GSLs in leaves. (A-B) Manhattan plots of GBS contents (A) and the corresponding QQ plot (B). (C-D) Manhattan plots of 4MeGBS contents (C) and the corresponding QQ plot (D). (E-F) Manhattan plots of 1MeGBS contents (E) and the corresponding QQ plot (F). GBS, Glucobrassicin; 4MeGBS, 4-Methoxyglucobrassicin; 1MeGBS, 1-Methoxyglucobrassicin.

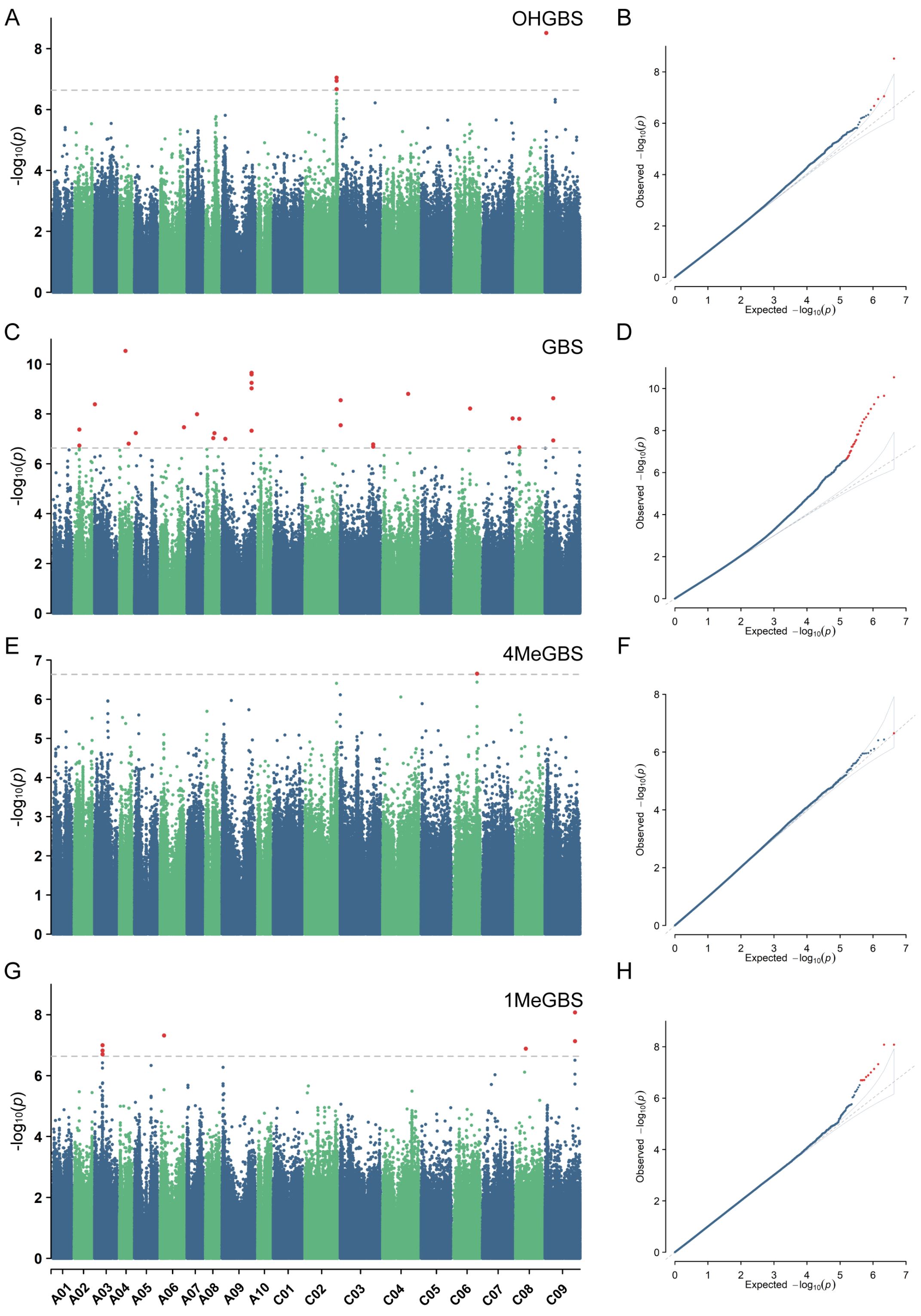

Supplementary Fig. S4. Genome-wide association study on indole GSLs in seeds. (A-B) Manhattan plots of OHGBS contents (A) and the corresponding QQ plot (B). (C-D) Manhattan plots of GBS contents (C) and the corresponding QQ plot (D). (E-F) Manhattan plots of 4MeGBS contents (E) and the corresponding QQ plot (F). (G-

H) Manhattan plots of 1MeGBS contents (G) and the corresponding QQ plot (H). OHGBS, 4-Hydroxyglucobrassicin; GBS, Glucobrassicin; 4MeGBS, 4-Methoxyglucobrassicin; 1MeGBS, 1-Methoxyglucobrassicin.
